# Chronothyroidology: Chronobiological Aspects in Thyroid Function and Diseases

**DOI:** 10.3390/life11050426

**Published:** 2021-05-10

**Authors:** Giuseppe Bellastella, Maria Ida Maiorino, Lorenzo Scappaticcio, Annamaria De Bellis, Silvia Mercadante, Katherine Esposito, Antonio Bellastella

**Affiliations:** 1 Unit of Endocrinology and Metabolic Diseases, Department of Advanced Medical and Surgical Sciences, University of Campania “Luigi Vanvitelli”, 80138 Naples, Italy; giuseppe.bellastella@unicampania.it (G.B.); mariaida.maiorino@unicampania.it (M.I.M.); lorenzo828@virgilio.it (L.S.); annamaria.debellis@unicampania.it (A.D.B.); 2 Diabetes Unit, Department of Advanced Medical and Surgical Sciences, University of Campania “Luigi Vanvitelli”, 80138 Naples, Italy; silvia.mercadante@studenti.unicampania.it (S.M.); katherine.esposito@unicampania.it (K.E.); 3 Department of Cardiothoracic and Respiratory Sciences, University of Campania “Luigi Vanvitelli”, 80138 Naples, Italy

**Keywords:** chronobiology, biological rhythms, thyroid diseases, rhythm disruption

## Abstract

Chronobiology is the scientific discipline which considers biological phenomena in relation to time, which assumes itself biological identity. Many physiological processes are cyclically regulated by intrinsic clocks and many pathological events show a circadian time-related occurrence. Even the pituitary–thyroid axis is under the control of a central clock, and the hormones of the pituitary–thyroid axis exhibit circadian, ultradian and circannual rhythmicity. This review, after describing briefly the essential principles of chronobiology, will be focused on the results of personal experiences and of other studies on this issue, paying particular attention to those regarding the thyroid implications, appearing in the literature as reviews, metanalyses, original and observational studies until 28 February 2021 and acquired from two databases (Scopus and PubMed). The first input to biological rhythms is given by a central clock located in the suprachiasmatic nucleus (SCN), which dictates the timing from its hypothalamic site to satellite clocks that contribute in a hierarchical way to regulate the physiological rhythmicity. Disruption of the rhythmic organization can favor the onset of important disorders, including thyroid diseases. Several studies on the interrelationship between thyroid function and circadian rhythmicity demonstrated that thyroid dysfunctions may affect negatively circadian organization, disrupting TSH rhythm. Conversely, alterations of clock machinery may cause important perturbations at the cellular level, which may favor thyroid dysfunctions and also cancer.

## 1. Introduction

From ancient hourglasses to sophisticated modern clocks, the measurement of time has been considered indispensable by man for the verification of the flow of natural and human phenomena. The earliest recorded recognition of the importance of biological rhythms in plants and animal dates back to at least 5000 BC. Light/darkness-correlated variations in leaf movements in some plants had been already observed by Androstene during the Empire of Alexander the Great, and the importance of temporal factors was even recognized in biblical times as stated in Ecclesiastes: “To everything there is a season and a time to every purpose under Heaven: a time to be born and a time to die; a time to plant and a time to harvest”. Moreover, as reported in Genesis, the light was created by God first of all, and this is of particular significance, since, as we will see later on, the alternation of light/darkness is the main synchronizing factor in the circadian rhythm [1]. The recent studies on the cyclic occurrence of several biological functions, and on the interrelationship between these functions and the cyclical variations of environmental factors, have allowed the foundation of a fairly new scientific discipline, chronobiology, which studies the time-related biological phenomena in relation to time, which assumes itself biological dignity. Considering that, among the time-related processes, there are some fundamental for life, such as birth, growth, sexual development and decline and ageing, and that some important physiological events, such as heart rate, blood pressure, coagulation and respiratory capacity, exhibit determined daily variations, we can understand the relevant importance of chronobiology in medicine [2,3]. In fact, the right key to reading the hormonal rhythmic variations of the human organism opens up new horizons for an early diagnosis of disorders of physiological chrono-organization, thus allowing the possible prevention of related clinical diseases and correct chronotherapy when they occur. Even the pituitary–thyroid axis is under the control of a central clock, and TSH secretion exhibits circadian, ultradian and circannual rhythms. This review aimed to highlight the main results from personal experiences and from other studies on this issue, which appeared in the literature as reviews, metanalyses and original and observational studies until 28 February 2021 and were acquired from two databases (Scopus and PubMed). After the description of the principal characteristics of biological rhythms, we deal with the chronophysiological aspects of hypothalamic–pituitary–thyroid axis secretions and then the chronopathological aspects of thyroid diseases.

## 2. Elementary Glossary of Chronobiology

Rhythmic activity is a fundamental property of living matter. It persists in constant environmental conditions because it is part of the genetic heritage of the species and therefore it has a hereditary character [4,5]. In humans, monozygotic twins present circadian rhythms with exactly overlapping characteristics, while individual differences may already be found in heterozygous twins, thus confirming that the individual expression of chronotype is strictly correlated to the individual’s genetic pattern [1]. The discovery and subsequent cloning of a hypothalamic biological “CLOCK” gene located at the suprachiasmatic nucleus (SCN) level, that dictates the timing of the rhythms from its site, and the description of other satellite genes that contribute in a hierarchical way to the regulation of the rhythms, have given the scientific seal to the assumption regarding the genetic organization of the rhythmic activity of the organism [2,6,7,8,9]. In particular, biological rhythms are regulated by transcription–translation feedback loops, which encode the molecular circuitry of circadian machinery, including several clock genes and proteins. The first step consists in the heterodimerization of CLOCK (circadian locomotor output cycles kaput) and BMAL1 (brain and muscle Arnt-like protein 1) to form a transcriptional activator complex, which gives a cascade of events at molecular levels involving several other genes, some activators, other repressors, leading to transcription–translation feedback loops of circadian clock genes, which drive the rhythmic expression of many satellite clock-controlled genes at the cellular level in central and peripheral organs, thus influencing in a time-correlated manner their physiological functions [2,6,7,8,9].

### 2.1. Main Characteristics of Biological Rhythms

The parameters of biological rhythms are the following: period, phase, mean level or mesor, amplitude [5,10].

*-Period* is the most important parameter of biological rhythms, as it identifies the time interval required to complete one cycle from the start to the end of the cycle, when it returns to the starting levels after having reached the maximum level (zenith or acrophase of the rhythm). According to the period, the rhythms are classified as follows: circadian (circa die: around one day), which indicates a rhythm with a period of around 24 h; ultradian, which indicates a rhythm at high frequencies with a period <24 h; infradian, which indicates a rhythm at low frequencies with a period >24 h. On the basis of their period, the infradian rhythms are classified as circaseptan, circatrigintan or circannual, for periods of around 7, 30 or 360 days, respectively. Many biological variables show circadian, ultradian and infradian oscillations, so it must kept in mind that circadian rhythms are only one frequency in the complex multifrequency human time structure [10,11].

*-Phase*, which is given by the value of a variable at a predetermined time with respect to the starting value.

*-Medium level or Mesor*, which represents the average value of a periodicity (for example, the average of the values of all samples taken for a given rhythm).

**Figure 1:**
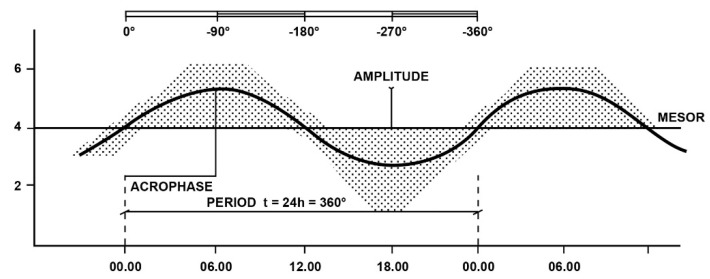
Schematic representation of a circadian virtual rhythm and its parameters (period, zenith or acrophase, mean level or mesor, amplitude), obtained by fitting a sinusoidal curve to a naïf pattern of data by the “least squares” method: Period of 24 h = 360° ([13], reproduced with permission of the Editor).

*-Amplitude*, which indicates the extent of a rhythm and is given by the maximum deviation of the variable from the middle level (at the upper limit: zenith or acrophase and at the lowest limit: nadir). Thus, it should not be confused with the macroscopic range of the rhythm, which is given by the difference between the highest and the lowest value of the rhythmic variable considered [5,10]. In Figure 1 is illustrated a schematic representation of a circadian virtual rhythm and its parameters (period, zenith or acrophase, mean level or mesor, amplitude), obtained by fitting a sinusoidal curve to a naïf pattern of data, by the “least squares” method [12,13].

### 2.2. Synchronizing Factors of Rhythms

Even if biological rhythms in mammals are genetically determined, they may be influenced by some endogenous and exogenous factors, which are able to strengthen or modify their characteristics. These factors are classified as “zeitgeber”, entraining agents or synchronizers [5]. The main synchronizing factors are summarized in Table 1.

**Figure 2:**
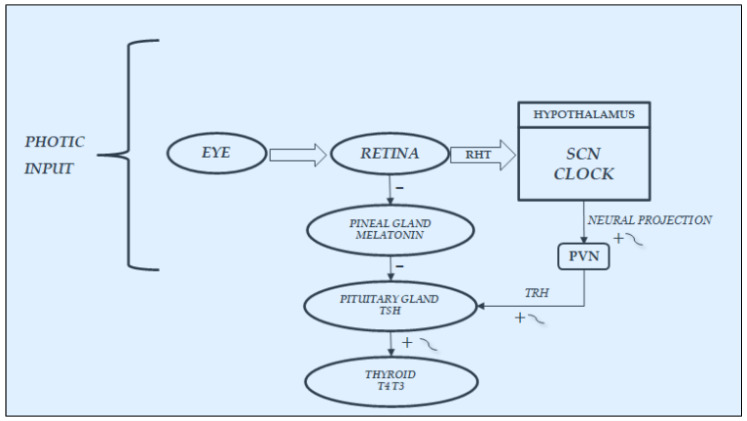
Synchronization by light input of the rhythmic variations in hypothalamic–pituitary–thyroid secretions: It acts directly, through the retino-hypothalmic tract (RHT), by exciting the light-entrainable circadian pacemaker located in the suprachiasmatic nucleus (SCN) of the hypothalamus, which then outputs the circadian signal via neural projection, exciting the rhythmic secretion of thyrotropin-releasing hormone (TRH) and consequently of thyrotropin (TSH) and thyroid hormones(T4:thyroxine; T3:triiodotyronine). Light acts also indirectly, by modulating with an inhibiting effect the variations in melatonin secretion at retina and pineal gland levels, thus further stimulating the secretions of the hypothalamic–pituitary–thyroid axis.

Even if these factors may not create rhythms, however, they may modify their parameters; thus, alterations of these schedules may cause a loss or change in normal circadian rhythmicity [5,10,13,14,15,16]. Among the exogenous ones, the light/dark cycle plays the most important synchronizing role in the endocrine rhythms, including those of the HPT axis. It plays this role directly, by exciting the light-entrainable circadian pacemaker located in the SCN of the hypothalamus [16,17], and indirectly, through variations in melatonin secretion [18,19]. In fact, the pineal gland plays an intermediate role in the variations in environmental factors, especially light and the endocrine and metabolic system, through variations in melatonin secretion, which is inhibited by light and stimulated by darkness, and, for this reason, it exhibits the highest value during the night [2,19] Since pineal melatonin exerts an inhibiting action on TSH pituitary secretion, the reduced pineal melatonin secretion induced by light stimuli indirectly favors an increase in TSH and thyroid hormone secretions (Figure 2).

Failure to comply with the correct light/dark alternation can negatively affect the metabolic processes of energy suppliers, favoring several diseases, including endocrine and metabolic ones [2]. Moreover, recent findings supported a link between circadian clocks, epigenetics and cancer [2,20]. Concerning the consequences of disruption of the circadian rhythmicity by an altered schedule of light/darkness and sleep/wake cycles, an example may be represented by epidemiologic data regarding female shift workers, demonstrating, among the risk for other diseases, an elevated cancer risk, especially for breast cancer [21]. Moreover, the pivotal role of light in clock function and in rhythm entrainment is also confirmed by data regarding blind subjects, especially those with blindness from birth, who suffer from sleep disorders and hormonal alterations, including thyroid dysfunction [18,22,23,24,25]. The correct knowledge of circadian variations in physiological variables and of pathological events may help the physician to make a better choice, not only for the prevention and diagnosis of diseases but also for planning a more effective chronotherapy. This will allow them, on the one hand, to restore normal rhythmicity, when previously altered, and, on the other hand, to achieve the maximum therapeutic result with the minimum side effects. A characteristic example of chronotherapy is represented by corticosteroid replacement therapy, which is usually carried out by administering the highest dose in the morning and a lower one in the afternoon or evening, or by administering a single morning dose of preparations that provide a time-related release in order to mimic the circadian rhythm of the hormone [13,14]. By considering the proper time of day for drug administration, we may optimize the therapeutic effect. An appropriate time-related therapy in hypercholesterolemic patients is obtained by administering short-acting statins in the evening, considering that the rate-limiting enzyme of cholesterol biosynthesis, 3-hydroxy-3-methylglutaryl coenzyme A reductase, peaks at night in humans [2]. Moreover, some chemotherapeutic agents increase their efficacy and reduce their toxicity in cancer patients when administered, taking into account the endogenous clock time [2,25,26]. In addition, alcohol toxicity differs according to the time of intake and increases when alcohol is consumed in the morning compared to evening intake [27].

## 3. Chronobiology and Hypothalamic–Pituitary–Thyroid Axis

### 3.1. Physiological Aspects

**Figure 3:**
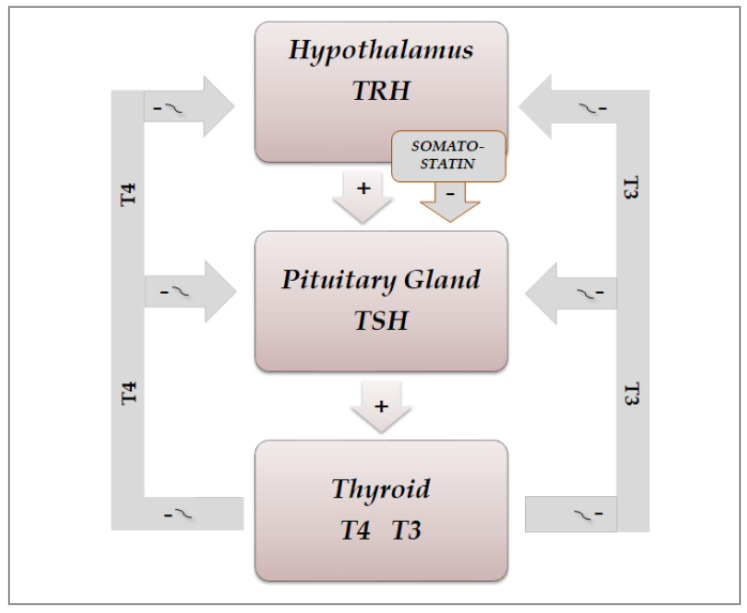
Schematic representation of physiological organization of rhythmic hypothalamic–pituitary–thyroid secretions: TRH(thyrotropin-releasing hormone) and somatostatin from the hypothalamus exert a stimulating and inhibiting action, respectively, on the pituitary secretion of TSH(thyrotropin), which in turn stimulates the thyroid gland to produce T4(thyroxine) and T3(triiodotyronine). Thyroid hormones regulate, by a negative feedback, the secretion of TRH and TSH, by acting both at hypothalamus and pituitary level.

The secretion of thyroid hormones, precursor T4 and active T3, is under the control of the hypothalamic–pituitary axis. Hypothalamic thyrotropin-releasing hormone (TRH) is a tripeptide secreted from the hypothalamic median eminence, which stimulates the release of thyrotropin (TSH), by reaching the anterior pituitary via the hypothalamic–pituitary portal system and binding to its membrane receptor on TSH-secreting cells located in the pars distalis of the gland [28]. Then, it stimulates the synthesis of TSH by inducing mRNA expression of TSHA and TSHB, which encode the TSH α and β subunits, respectively. Chemically, the pituitary hormone TSH is a non-covalently linked heterodimer glycoprotein consisting of the two subunits, which stimulates the thyroid gland to produce T4 and T3 by binding to its receptor, a G protein-coupled receptor, located on the thyroid follicle membrane, which in turn stimulates the secondary messengers cAMP and inositol phosphate [29,30]. Figure 3 shows the secretions of the hypothalamic–pituitary–throid axis. Thyroid hormones regulate, by a negative feedback, the secretion of both TRH and TSH, by acting at hypothalamic and pituitary level, respectively. A further direct inhibiting action on TSH secretion is exerted by somatostatin, through binding to four of the five receptor subtypes (SSSTR1, 2, 3 and 5), which are expressed at pituitary level also on TSH-secreting cells, while SSTR2 and 5 are the most abundantly expressed on somatotrophs [31,32].

Thyroid hormones circulate while bound reversibly to three types of proteins: thyroxin-binding globulin (TBG), transthyretin, and albumin. Only 0.02% of T4 and 0.3% of T3, known as free T4 and free T3, are unbound and are the active hormones, which are transported into target tissues by membrane transporters [33].

### 3.2. Chrono-Organization of Hypothalamic–Pituitary–Thyroid Axis

#### 3.2.1. Physiological Aspects

**Figure 4:**
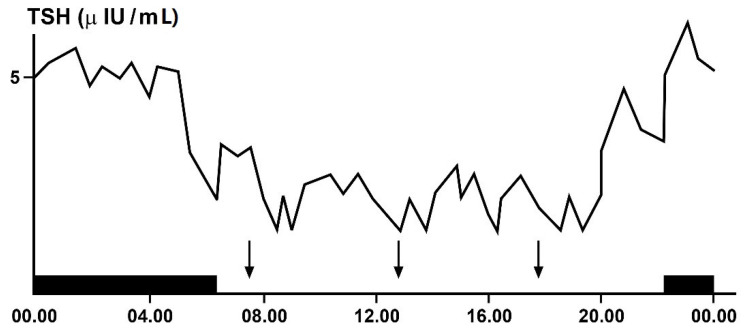
Circadian and ultradian variations in plasma TSH (thyrotropin)concentrations obtained by frequent samples over 24 h in a single healthy adult volunteer. The black bar indicates the period of sleep, the arrows the time of the meals.

Even hypothalamic–pituitary–thyroid (HPT) axis secretions, as already noted, are under the control of the suprachiasmatic nucleus (SCN) pacemaker and exhibit rhythmic oscillations. Studies in animals clarified the neural and molecular mechanisms underlying the circadian regulation of the HPT axis, also revealing the unexpected role of TSH and thyroid hormones in the seasonal regulation of reproduction [30,34,35,36,37]. The results of some of these studies demonstrated a functional connection between the SCN and the thyroid gland, revealed by thermic ablation of this nucleus and by viral tracing techniques in the rat [37]. The thermic ablation of the biologic clock at SCN level eliminated the diurnal oscillations of TSH and thyroid hormones but with a more pronounced effect on thyroid secretions, as opposed to TSH secretion. Moreover, retrograde virus tracing, used to identify the type and localization of neurons in the central nervous system involved in the control of the autonomic innervations of the thyroid gland, showed that the virus infected, among several neurons in the brain, even the paraventricular nucleus of the hypothalamus, including TRH-containing cells and several other hypothalamic structures, such as the SCN. On the basis of their results, the authors proposed a dual control mechanism for thyroid function by SCN: on the one hand, by affecting the neuroendocrine control of TSH release and, on the other hand, by controlling the autonomic input directly to the thyroid gland [37]. TSH secretion shows a circadian and ultradian (pulsatile) rhythmicity with an increasing concentration from the late afternoon or early evening before sleep onset and a peak in secretion during the early part of the night. Subsequently, TSH secretion declines during the sleep period until it reaches low daytime levels [30]. In fact, sleep seems to exert an inhibitory effect on nocturnal TSH secretion: patients subjected to sleep deprivation do not show a significant decrease in TSH concentrations [30,35,36]. The inhibiting effect of sleep on TSH secretion has further been confirmed by the finding of increased nocturnal TSH levels in patients who carry out night-shift work [38] (Figure 4).

#### 3.2.2. Pathophysiological Aspects

Other pathophysiological conditions, such as obesity, age, and longevity, may affect the daily oscillations of TSH [39]. Moreover, the elevated secretion of cortisol in patients with Cushing’s syndrome has been seen to abolish the nocturnal serum TSH surge [40]. Moreover, TSH oscillations in patients with type 1 diabetes may be directly affected by glycemic excursions, regardless of variations in thyroid hormone concentrations: in fact, an inverse correlation was observed between glycemic values and TSH levels but no correlation was found between glycemic values and thyroid hormones [41].

Among the exogenous factors which can affect thyroid function, the effect of magnetic fields is still discussed. A circadian study performed some years ago on the secretions of pituitary, thyroid, and adrenocortical hormones in young men exposed for one night to a 50 Hz magnetic field did not evidence significant alterations of the hormonal secretions of these glands or of their circadian rhythmicity [42]. Instead, a more recent study on the effects of electromagnetic radiation exposure on bone mineral density, thyroid metabolism, and oxidative stress in electrical workers demonstrated that this exposure may affect all the variables investigated [43].

### 3.3. Light/Darkness and Thyroid Function

As already noted in previous paragraphs and depicted in Figure 2, an important stimulatory and synchronizing role in the secretions of HPT axis is played by light stimuli, which favors the secretions of this axis, on the one hand, arriving from the retina directly to the SCN-Clock site, through the retino-hypothalamic trait (RHT) and, on the other, reaching the pineal gland and inhibiting melatonin secretion. In fact, findings in laboratory animals have demonstrated that melatonin can play an inhibitory role in the secretions of the HPT axis; thus, light stimuli, through the inhibition of its secretion, may favor the function of this axis [10,44,45,46,47,48]. In humans, blind subjects may be considered a natural, experimental, although unlucky, model to study the effect of light on the endocrine system [18,22,24,49]. In fact, the lack of light stimulus increases melatonin secretion at pineal level but also directly at retina level, considering that the sensory receptors of the retina can be considered a special class of hormonal cells, as studies in animals demonstrated that they are able to synthesize melatonin directly [50,51], also exhibiting the circadian rhythmicity of this hormone when cultured in vitro [52]. Moreover, the hormonal secretions in blind subjects, including those of the HPT axis, are further impaired not only by the loss of the synchronizing effect of light stimuli on the SCN-Clock but also by the lack of the light action on the sensory receptors of the retina. In fact, these retinal structures are able to produce not only melatonin [50,51] but also TRH, which could contribute, together with the hypothalamic TRH secretion, to stimulate pituitary TSH secretion [45]. Considering that light stimuli inhibit melatonin secretion and stimulate TRH secretion also at retinal level, the lack of these stimuli in blind subjects, on the one hand, induces an increase in melatonin and, on the other hand, causes a reduction in TRH production, with consequent further impairment of the HPT axis secretions.

However, the effect of blindness on the hormonal secretions of the HPT axis through melatonin variations seems to be different before and after puberty [53,54]. Several years ago, we studied the thyroid function in young blind males aged 7–10 yr, in Tanner stage one puberty, living at the “Martuscelli” Institute for young blind subjects, in Naples, Italy [53]. Each had a TRH test at 08.00 h after nocturnal rest. Plasma TSH, total and free T3, total and free T4, and cortisol were measured by RIA. Our blind subjects showed normal levels of basal and TRH-stimulated TSH, T4, and T3, but FT3 and FT4 were significantly higher than controls. We concluded that our results, similar to those usually found in some patients with euthyroid hyperthyroxinemia [54], suggested that the prolonged inability to receive light signals could influence the metabolism of thyroid hormones and/or cause an acquired peripheral and pituitary tissue resistance to their action similar to the familial syndrome of thyroid hormone resistance described by Refetoff and coworkers [55,56]. To clarify whether the abnormality found in our young blind patients persisted after puberty, we studied thyroid function eight years later (TSH, total and free T3 and T4, reverse T3, TBG, and melatonin on plasma samples drawn at 08.00 a.m. after nocturnal rest) in blind adults, living at the “Colosimo” Institute for blind adults in Naples, Italy. Surprisingly, blind adults did not show hormonal values significantly different from those of controls, except for significantly higher melatonin levels. We concluded that a possible resetting of the HPT axis after puberty could occur in blind subjects, hypothesizing that variations in melatonin secretion could play a role in this resetting [57]. However, our assumption was speculative, because melatonin had not been investigated in our prepubertal blind subjects and a possible resetting role exerted by the increase in gonadal hormone secretion in adult blind patients could not be excluded. Even if our studies did not investigate the circadian variations in the hormones but only the morning basal concentrations, however, they further testify to the importance of light stimuli in the hormonal secretions of the hypothalamic–pituitary–thyroid axis. This is also considering that this stimulus arrived at the SCN through RHT and that many TRH-containing cells were evidenced by immunocytochemistry in human brain material obtained with a short postmortem delay, and particularly in the paraventricular nucleus (PVN) and in SCN, the site of the circadian clock of the brain, which regulates, among other variables, the rhythmic secretions of the HPT axis [58].

### 3.4. Ultradian Rhythms of TSH and Thyroid Hormones

Pituitary hormones, including TSH, and thyroid hormones show also ultradian and infradian rhythmicity [10,11,30,59]. An ultradian rhythm of TSH has been demonstrated in humans with a short period of around 30 min and low amplitude, reflecting a pulsatile release of this hormone [30,60], strictly influenced by thyroid and adrenal hormones [40] and also by the sleep stage, such as slow-wave sleep [36]. Moreover, a decreased pulse amplitude of the TSH ultradian oscillations has been observed during fasting [61].

### 3.5. Infradian Rhythms: Seasonality of HPT Axis Secretions

Studies in animals evidenced that TSH and thyroid hormones may follow a seasonality. Their seasonal variations are in some way related to the seasonal changes in some physiological processes, such as reproduction, migration, and hibernation [30]. Infradian variations in these hormones occur also in humans, even if the occurrence of circannual rhythms of TSH and thyroid hormones is still under discussion [30]. Studies in adult males living in the Netherlands reported that plasma thyroid hormone concentrations decrease during the summer, being inversely correlated with the seasonally altering environmental temperature [30,62]. Instead, studies investigating the circannual pattern of HPT function and mood in subjects of both sexes, during extended Antarctic residence, showed that both TSH and mood exhibited a circannual pattern, with peaks during the months of November and July and a trough during the months of March and April. A decline in free T3 and T4 preceded high levels of mood disturbance; however, increases in tension-anxiety preceded a decline in free T3 levels, suggesting a feedback of mood on T3 but not on T4 levels [63].

**Figure 5:**
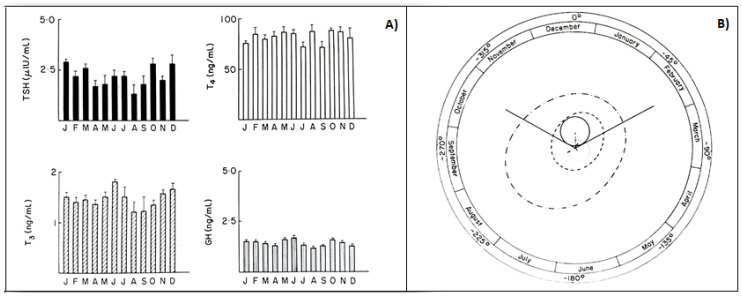
(**A**) Monthly mean (±SD) of TSH, T4, T3, GH values in prepubertal subjects. (**B**) Single cosinor display of circannual variations in these hormone concentrations and their characteristics, visualized by polar cosinor plot. A significant rhythm was validated only for TSH, as revealed by its ellipse (continuous tract), which does not cover the pole (zero: center), and with acrophase in December, as indicated by the arrow starting from the center and directed towards December (confidence limits October–February) [68].

In animals, phenotypic flexibility is initiated before birth and is linked to the pattern of photoperiod exposure experienced by the mother during pregnancy. The maternal photoperiod programs the hypothalamic thyroid status of offspring through transplacental communication via the pineal hormone melatonin, thus conditioning their chrono-organization already at birth [64]. The role of melatonin is further confirmed by variations in thyroxin secretion after pinealectomy [65]. A similar mother–offspring transmission occurs also in humans, as the rhythmic secretions of the HPT axis, including the infradian ones, are present in young subjects already before puberty. In fact, children with or without goiter showed seasonal variations in total and free thyroid hormones, with the highest values in the fall, and in TSH, with the highest values in summer [66]. Several years ago, to clarify the occurrence of circannual GH, TSH, T4, and T3 rhythms in prepuberty, we had been studying, for a four-year period, the seasonal variations in these hormones in 150 healthy subjects, aged 6–10. The occurrence of any circannual rhythm was statistically investigated by the cosinor method [5,12]. A significant circannual rhythm was validated only in TSH secretion, with annual crest time in December, while GH, T4, and T3 did not show a circannual rhythm (Figure 5). We concluded that thyroid hormones, at least before puberty, do not play a pivotal role in the regulation of circannual TSH periodicity [67].

In the subsequent years, we studied whether the occurrence of a circannual TSH rhythm was present also in healthy adult men and whether the genetic and hormonal alterations occurring in Klinefelter’s syndrome were responsible for abnormalities of this rhythm in affected patients [68]. For this purpose, we had been investigating for 3 years the monthly TSH variations in 73 patients affected by Klinefelter’s syndrome and 69 healthy adult males, matched for age and weight. The occurrence of any circannual rhythm was statistically investigated by the cosinor method. Healthy adults showed a significant circannual TSH rhythm with acrophase in December, as previously observed in prepubertal boys, while patients with Klinefelter’s syndrome showed circannual mean TSH levels significantly lower than controls without any statistically significant circannual TSH rhythm [68]. We concluded that, in this syndrome, an abnormality in hypothalamic–pituitary coordination impairing TSH release could also involve circannual periodicity of this hormone and, thus, with respect to clinical implications, the treatment of patients with this syndrome should be also aimed at restoring the lost circannual rhythmicity [68]. A circannual rhythm of TSH with the same characteristics found in our prepubertal and adult healthy subjects has recently been confirmed in Japanese people with the highest concentrations in winter, disengaged from thyroid hormone variations but inversely related to seasonal variations in environmental temperature [69]. Considering that studies performed in animals evidenced an unexpected role of TSH in regulating seasonal processes related to reproduction [34] and that a similar role could also be played in humans, it is advisable to take great care to prevent disruption of the chrono-organization of the HPT axis to protect their reproductive lives.

## 4. Interrelationship between Disorders of Chrono-Organization and Thyroid Diseases

### 4.1. Autoimmune Thyroid Diseases and Circadian System

The most frequent thyroid diseases causing thyroid dysfunction, both hyper- (Graves disease) and hypothyroidism (Hashimoto thyroiditis), are triggered by an autoimmune process involving the production of autoantibodies, which impairs thyroid function. These antibodies, in the first case (TR-Ab), cause hyperthyroidism, by stimulating directly the thyroid to produce elevated amounts of T4 and T3, regardless of variations in TSH concentrations, which remain constantly at a low level. Instead, in the second case (TgAb and TPOAb), they act at thyroid level, by inhibiting the secretion of these hormones and causing hypothyroidism and, as a rebound effect, an increase in TSH concentrations.

Several studies investigated the relationship between the immune system and circadian machinery, highlighting the negative effect of circadian disruption on this system [70,71,72]. In particular, a combined influence of the circadian system and sleep may induce an increase in circulating naïve T-cells and the production of some proinflammatory cytokines, such as interleukin-12, during nighttime, and that of cytotoxic effector leukocytes and of interleukin-10 during daytime, with strong clinical implications [70]. Moreover, chronic sleep deprivation or restriction desynchronizes central and peripheral clocks and impairs the immune response by disrupting circadian rhythms at the level of immune cells and, through this mechanism, deregulates the immune system [71]. Recent studies have clarified the molecular mechanisms by which the circadian clock controls the immune system. This control is exerted through the involvement of circadian clock proteins acting as transcription factors, driving the expression or repression of immune genes. It moreover involves the acetylation or methylation of histones to regulate gene transcription or inflammatory proteins. On the other side, circadian clock proteins can engage in direct physical interactions with some components of key inflammatory pathways, allowing the immune system to reciprocally exert control over circadian clock function [72]. Patients have to be informed on the uncorrected behaviors which can alter their normal circadian rhythmicity, to avoid the disruption of the virtuous interconnection between the circadian machinery and the immune system that may favor the occurrence of immune diseases, including those involving the thyroid gland.

### 4.2. Thyroid Dysfunctions and Circadian Clock

Both hypothyroidism and hyperthyroidism alter the circadian clock. Clinical or subclinical primary hypothyroidism may affect rhythmic TSH oscillations differently. Patients with clinically overt hypothyroidism have impaired levels of free thyroid hormone, with TSH concentrations that are markedly high, which usually obliterate the detection of the daily secretion of the hormone, whereas patients with subclinical hypothyroidism, in whom free thyroid hormones are still in the normal range and TSH levels are slightly increased above the normal range, may show a daily secretion pattern of the hormone that is still sustained [30,39,73]. Patients with central hypothyroidism, especially if caused by TRH deficiency or cranial irradiation for brain malignancies, show disruption or absence of TSH rhythm [30], whereas those with pituitary resistance to thyroid hormone action due to defects of thyroid hormone receptors may show a circadian rhythm of TSH concentrations that are still preserved [74].

With regard to the effects of hyperthyroidism on the circadian rhythmicity, we must take into account the three conditions responsible for hyperthyroidism, i.e., first of all, the presence of immunoglobulins that bind to the TSH receptor, as in Graves’ disease, causing overstimulation of the thyroid gland, then the activating mutations of the TSH receptor, and, more rarely, pituitary TSH-producing tumors. In the first two conditions, TSH is suppressed and its circadian rhythmicity is blunted, whereas in the third case, the diurnal TSH rhythm, even if, with a secretory pattern, more irregular and with increased pulse frequency, may be preserved [30,75]. Recent studies in hypothyroid and hyperthyroid animals have elucidated the molecular mechanisms by which variations in thyroid hormone levels interact with circadian gene expression [73]. In particular, hypothyroidism has been shown to disrupt the circadian expression pattern of brain and muscle Arnt-like protein-1 (BMAL1) and of period circadian regulator 2 (Per2) and to decrease the mesor of nuclear receptor subfamily 1 (Nr1d1) and of thyrotropic embryonic factor (TEF). Instead, hyperthyroidism increases the mRNA expression of core clock genes and TEF, as well as the mesor and amplitude of BMAL1 and the mesor of Nr1d1. The disruption of the circadian expression of these genes by hypothyroidism and the increased expression induced by hyperthyroidism at pituitary level are responsible not only for disorders of daily secretion of TSH but also of disorders of other pituitary hormones [73].

### 4.3. Circadian Clock and Thyroid Malignancy

Recent studies revealed strong changes in clock gene expression in various types of human cancer, including thyroid cancers [2,20,30,76,77,78,79,80]. Characterization of the thyroid clock machinery alterations upon thyroid nodule malignant transformation have contributed to clarification of the connections between circadian clocks and thyroid cell oncogenic transformation [76,78,79]. The study of clock transcript levels by quantitative RT-PCR in thyroid tissues obtained by biopsies of normal and nodular thyroid tissue evidenced different expression levels of some clock genes in malignant thyroid cells with respect to thyroid normal cells. In particular, the expression levels of BMAL1 were upregulated and those of Cry2 were downregulated in samples of follicular and papillary thyroid carcinoma, as compared with normal thyroid and benign nodules. This contributed to an understanding of the connections between circadian clocks and oncogenic transformation of thyroid cells [76]. Studies in Zebrafish models on the role of circadian rhythms and hypoxia in cancer and metastasis demonstrated that pathological tumor blood vessels cause hypoxia and disruption in circadian rhythmicity, which in turn drives tumor metastasis [77]. The biological link between circadian clockwork disruption and thyroid tumorigenesis has been further highlighted by a more recent paper, which has also pointed out the potential clinical implications of this link and of its impact on thyroid cancer prevention, diagnosis, and therapy [80]. Moreover, considering, in particular, the important role played by TEF in suppressing malignant cells and tumorigenesis [81], the disruption of circadian machinery involving this factor could also favor an increase in thyroid malignancy.

Focusing the attention on the desynchronizing factors of HPT axis machinery, disruption of plasma levels of TSH and T3 has been observed in those affected by shift work, jet lag, and chronic sleep disorders [30]. In particular, disorders of sleep/wake and light/darkness rhythms, such as also occurring, for example, in nighttime work, are associated with a higher incidence of some cancers [20,21,82,83]. The findings regarding the association between light at night, melatonin secretion, sleep deprivation, and disruption of internal clock have been extensively reviewed by Touitou and coworkers, who examined even the proposed countermeasures to the effects of these desynchronizing events [82]. In fact, a possible prevention or a prompt correction of the desynchronizing effects of these events may be advisable to avoid more important consequences for human health. In this context, emerging evidence indicates that correct chronotherapy by synchronizing drug delivery with the endogenous physiological rhythm may be used to optimize treatment efficacy. In fact, adjusting the administration of some drugs to the time has been shown to improve their therapeutic effects and reduce off-target side effects [2,25,26,27]. It is more difficult to therapeutically restore a normal rhythmicity once it is disrupted. Some hormones and drugs are tested for this purpose, to investigate whether they can play a chronobiotic effect. One of these drugs may be considered melatonin, which, when administered in the evening, is able to reset the sleep/wake rhythm that was previously desynchronized from 24 h schedules, and when taken before a trans-oceanic journey, it may abolish or considerably attenuate the jet lag syndrome [2,10,13,19]. Moreover, among the hormones, Ghrelin has been shown to have chronobiotic characteristics. In fact, treatment with this hormone of cultured hepatocytes from steatotic liver was able to markedly restore the circadian pattern of clock genes such as Bmal1 and Per, previously blunted by steatosis [83]. Considering that these genes are involved in the transcriptional–translation feedback loop, it should be advisable to investigate in humans whether a possible use of Ghrelin or other chronobiotic therapeutic factors may restore circadian disruption and avoid its related pathological consequences, including cancer.

## 5. Conclusions

Knowledge and respect of biological rhythms and of their endogenous and environmental synchronizing factors is mandatory not only for physicians but also for patients, to ensure the best conditions for the lives of human beings. This is particularly important for the HPT axis, as the knowledge and respect of the reciprocal relationship between the secretions of this axis and the circadian machinery may avoid, on the one hand, the disruption of the circadian rhythmic organization of the subjects and, on the other hand, the consequences of this disruption, which can cause not only thyroid dysfunction but also thyroid cancer. Further studies have to be encouraged to better clarify physiological and pathophysiological aspects of this issue and to search for more appropriate preventive choices to avoid circadian but also ultradian and infradian rhythm disruption of the HPT axis and to search for more effective therapeutic options to promptly correct these alterations, when they have already occurred.

## Figures and Tables

**Table 1:** Main synchronizing factors of biological rhythms.

Light/darkness cycle
Sleep/wake alternations
Periodic food intake
Social environment
Physical and mental work
Appropriate energy variability
Individual chronotype

## Data Availability

Not applicable.
